# Evaluation of serum MMP-9 as predictive biomarker for antisense therapy in Duchenne

**DOI:** 10.1038/s41598-017-17982-y

**Published:** 2017-12-20

**Authors:** A. Lourbakos, N. Yau, P. de Bruijn, M. Hiller, K. Kozaczynska, R. Jean-Baptiste, M. Reza, R. Wolterbeek, Z. Koeks, B. Ayoglu, D. de Klerk, G. Campion, I. Zaharieva, V. D. Nadarajah, P. Nilsson, C. Al-Khalili Szigyarto, F. Muntoni, H. Lochmüller, J. J. Verschuuren, N. Goemans, M. Tulinius, E. H. Niks, S. de Kimpe, A. Aartsma-Rus, Peter A. C. ’t Hoen, P. Spitali

**Affiliations:** 1 BioMarin Nederland BV, J.H. Oortweg 21, 2333 CH Leiden, The Netherlands; 2 Department of Human Genetics, Leiden University Medical Center, PO Box 9600, 2300 RC Leiden, The Netherlands; 3 John Walton Muscular Dystrophy Research Centre, Institute of Genetic Medicine, Newcastle University, Newcastle upon Tyne, UK; 4 Department of Medical Statistics, Leiden University Medical Center, PO Box 9600, 2300 RC Leiden, The Netherlands; 5 Department of Neurology, Leiden University Medical Center, PO Box 9600, 2300 RC Leiden, The Netherlands; 6 Department of Proteomics, School of Biotechnology, KTH-Royal Institute of Technology, Stockholm, Sweden; 7 Dubowitz Neuromuscular Centre and MRC Centre for Neuromuscular Diseases, UCL Institute of Child Health, London, UK; 8 Child Neurology, University Hospitals Leuven, Leuven, Belgium; 9 Department of Pediatrics, University of Gothenburg, Queen Silvia Children’s Hospital, Gothenburg, Sweden

## Abstract

Duchenne Muscular Dystrophy (DMD) is a severe muscle disorder caused by lack of dystrophin. Predictive biomarkers able to anticipate response to the therapeutic treatments aiming at dystrophin re-expression are lacking. The objective of this study is to investigate Matrix Metalloproteinase-9 (MMP-9) as predictive biomarker for Duchenne. Two natural history cohorts were studied including 168 longitudinal samples belonging to 66 patients. We further studied 1536 samples obtained from 3 independent clinical trials with drisapersen, an antisense oligonucleotide targeting exon 51: an open label study including 12 patients; a phase 3 randomized, double blind, placebo controlled study involving 186 patients; an open label extension study performed after the phase 3. Analysis of natural history cohorts showed elevated MMP-9 levels in patients and a significant increase over time in longitudinal samples. MMP-9 decreased in parallel to clinical stabilization in the 12 patients involved in the open label study. The phase 3 study and subsequent extension study clarified that the decrease in MMP-9 levels was not predictive of treatment response. These data do not support the inclusion of serum MMP-9 as predictive biomarker for DMD patients.

## Introduction

Duchenne muscular dystrophy (DMD) is a neuromuscular disorder with an incidence of 1 in 5000 newborn males^1^. DMD is caused by the absence of dystrophin due to protein truncating mutations in the *DMD* gene^2^. In-frame mutations in the same gene allow formation of partially functional dystrophin and cause the milder Becker muscular dystrophy (BMD)^3^. There is no cure available for DMD but the standard of care includes use of anti-inflammatory corticosteroid drugs such as prednisone and deflazacort, which are capable of delaying disease progression^4^. In the last 15 years several therapeutic approaches have been developed following experimental evidence in cells and in animal models, showing it was possible to restore dystrophin, improve muscle quality, reduce oxidative stress and delay disease progression^5–14^. Some compounds have been tested in clinical trials where groups of patients were treated with different dosages or treatment regimens^15^. Antisense oligonucleotides (AONs) mediated exon skipping restored dystrophin in DMD patients after intramuscular and systemic delivery with two independent chemistries and the morpholino compound has recently received accelerated approval by the FDA^16–20^. Due to the mutation-specific nature of the exon skipping approach, only subsets of patients are eligible for a clinical trial assessing the skipping of a certain exon, leading to AON trials generally being conducted in small cohorts of patients. Also the low potency of the exon skipping drugs currently tested in patients calls for longer observational time to avoid that treatment-induced changes could be masked by inter-patient variability. The lack of objective functional outcome measures increases this risk even further. At the moment, the functional measure used in clinical trials for DMD is the 6 minute walk test (6MWT)^21^, which can only be performed in ambulatory patients (the majority of patients is non-ambulant) and where the outcome has been shown to be influenced by patient’s motivation. The identification of functional and molecular outcome measures able to monitor disease progression and predict response to treatment is a priority for the field, also to avoid invasive muscle biopsies which constitute a burden for patients and their families^22^. The only diagnostic serum biomarker available at the moment is muscle creatine kinase, the serum levels of which are elevated in DMD patients and similarly elevated in BMD. Unfortunately, serum creatine kinase concentration is also strongly influenced by exercise and decreases at the later stages of the disease due to loss of muscle tissue^23^.

Previously, we reported serum levels of matrix metalloproteinase-9 (MMP-9) to be elevated in DMD patients over healthy controls and to increase over time in 9 DMD patients^24^.

The overall objective of this study was to assess whether MMP-9 serum levels increases over time in DMD patients and to provide proof that it could be used as exploratory biomarker in clinical trials aiming to restore dystrophin. Towards this goal we (i) used 3 independent techniques to validate the association of MMP-9 serum levels with Duchenne, (ii) we characterised the MMP-9 isoforms in DMD by gelatin zymography, (iii) we confirmed in 2 large longitudinal cohorts that MMP-9 levels increase over time and (iv) we studied MMP-9 levels in two clinical trials involving patients treated with drisapersen, a 2′-*O*-methyl-phosphorothioate antisense oligonucleotide able to skip exon 51 in the dystrophin pre-mRNA.

## Results

### Serum MMP-9 levels are higher in DMD patients compared to healthy controls

**Figure 1:**
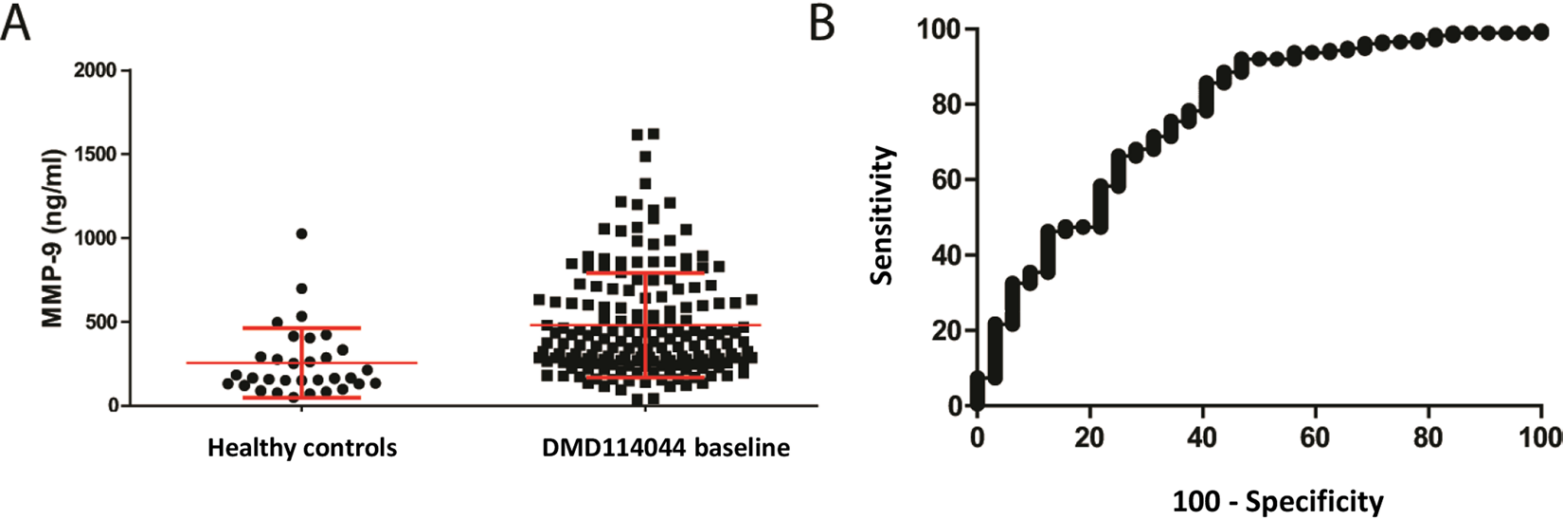
MMP-9 serum levels are elevated in DMD. (**A**) Dot plot showing the distribution of MMP-9 serum levels in 32 healthy controls and 172 DMD patients. Samples of DMD patients represent the baseline of the phase 3 DMD114044 study. (**B**) ROC curve showing the potential of MMP-9 serum levels to discriminate between DMD patients and healthy (age-matched) controls.

Serum MMP-9 levels were found to be elevated in DMD sera compared to controls^24^ (and Spitali *et al*., submitted, and Table S1). MMP-9 serum levels were quantified in 32 healthy controls and 172 DMD patients involved in the phase 3 trial (DMD114044). Mean MMP-9 levels were 256.7 ng/ml for the healthy group and 479.8 ng/ml for the DMD group, highlighting a statistically significant difference between the groups (p < 0.0001, Fig. 1A). ROC curves analysis exhibited an area of 0.77, corresponding to a good but not excellent capacity to discriminate between DMD and healthy (Fig. 1B). Since MMP-9 levels have been found to be elevated in serum compared to plasma due to MMP-9 release during clotting^25^, we studied MMP-9 in 111 DMD individuals participating to the natural history study for whom serum and plasma was obtained at the same moment. A highly significant correlation was found between serum and plasma MMP-9 levels using both ELISA and antibody array (Fig. S1A–D). Gelatin zymography clarified that elevated MMP-9 levels in DMD were imputable to the precursor form of MMP-9 (Fig. S1E–F).

### Serum MMP-9 levels increase over time in DMD patients

**Figure 2:**
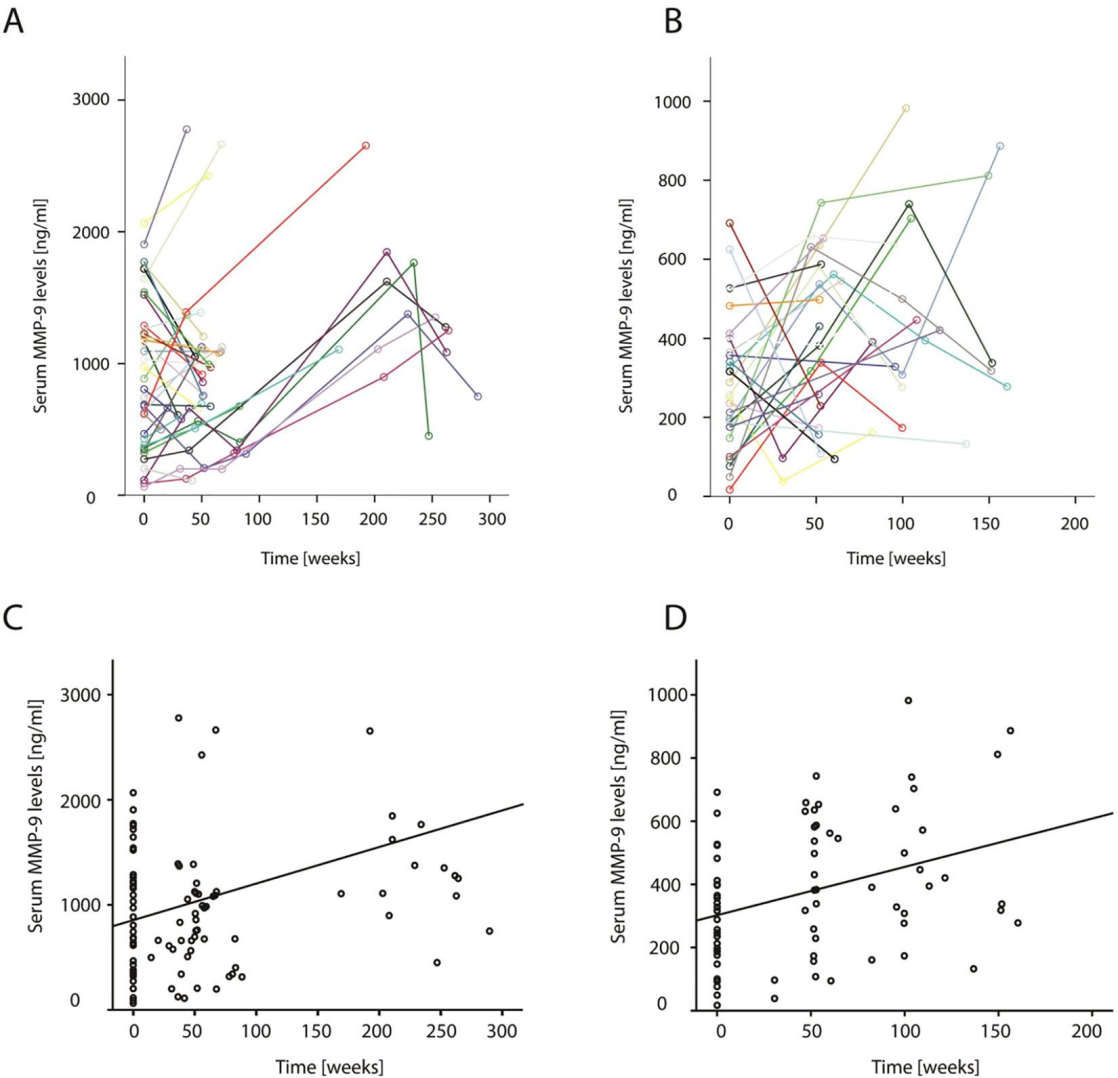
Serum MMP-9 levels increase over time in DMD patients’ serum samples. (**A**,**B**) Scatter plots showing serum MMP-9 concentration (y-axis) with follow-up time (x-axis) in individual DMD patients from NCL (**A**) and LUMC (**B**). Colors represents specific patients and color matched lines connect longitudinal measurements. (**C**,**D**) Scatter plots representing the same data as in panels A and B. In these 2 panels the line represents the time effect as estimated by the fixed effects of the linear mixed model. The increase of MMP-9 levels over time is significant (p < 10^−7^ for NCL and p < 10^−2^ for LUMC).

We collected longitudinal samples from patients participating in natural history studies in Leiden and Newcastle to clarify whether serum MMP-9 levels change over time as the disease progresses. MMP-9 levels at baseline were estimated 242.3 ng/ml and 294.4 ng/ml for NCL and LUMC cohorts, respectively. Age at baseline was significantly associated with MMP-9 levels in the NCL cohort (p < 10^−3^), while this association was not significant in the LUMC cohort. Follow-up time was significantly associated with MMP-9 serum levels in both cohorts (p < 10^−7^ for NCL and p < 10^−2^ for LUMC) and was estimated to increase by 3.5 ng/ml per week in the NCL cohort and by 1.5 ng/ml per week in the LUMC cohort (Fig. 2). The number of patients and corresponding longitudinal samples in these cohort is reported in Table S2.

### Serum MMP-9 levels are not affected by treatment with drisapersen

**Figure 3:**
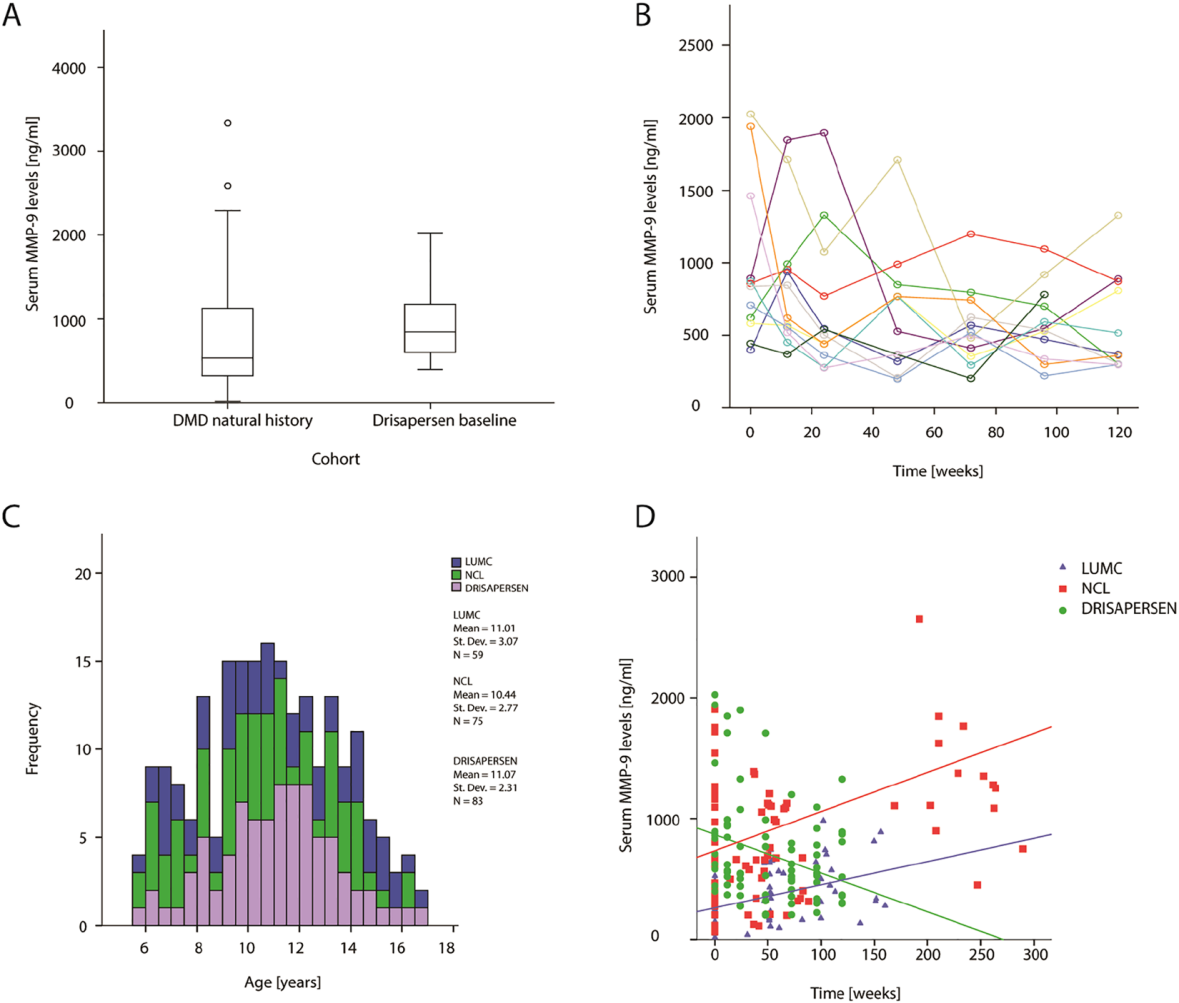
Serum MMP-9 levels in patients participating to the dose escalating study (NCT01910649) and open label extension study with drisapersen (study 114673). (**A**) Box plots show comparable serum MMP-9 concentration in DMD natural history cohorts (patients belonging to the 3 natural history cohorts pooled together) and the baseline visit of DMD patients treated with drisapersen. (**B**) Scatter plot representing serum MMP-9 concentration (y-axis) with follow-up time (x-axis) in 12 DMD patients treated with drisapersen. MMP-9 levels significantly decrease over time (p < 10^−3^). (**C**) Histogram showing the age distribution of the 3 longitudinal cohorts. Mean, standard deviation and counts are reported for each cohort. (**D**) Scatter plots showing the increase of MMP-9 levels over time in the 2 natural history cohorts and the decrease of MMP-9 in the drisapersen treated cohort. All longitudinal samples in the same age range are included in this graph. Each dot represents a longitudinal measurement. Colors are specific for cohorts. Lines represent follow-up time as estimated by the fixed effects of the simplified linear mixed model only including follow-up time.

To evaluate whether serum MMP-9 levels could not only monitor disease progression but also response to treatment, we measured MMP-9 levels in 12 patients participating in an open label extension trial where DMD boys amenable to exon 51 skipping were treated with 6 mg/kg/wk of drisapersen. Figure S2 shows the distribution of MMP-9 serum levels in the drisapersen treated cohort from the start of the dose escalation and during the open label extension study. Serum MMP-9 concentrations at baseline (start of extension study) were comparable with levels measured in the natural history cohorts (Fig. 3A). Analysis of the drisapersen longitudinal samples showed a significant reduction of MMP-9 serum levels over time by 3.2 ng/ml per week (p < 10^−3^) (Fig. 3B). To test differences among longitudinal cohorts we selected DMD patients from the natural history cohorts of LUMC, NCL who were in the same age range as drisapersen treated patients (Fig. 3C). Mean age and age range were comparable between LUMC, NCL and drisapersen cohorts. The increase of MMP-9 over time in the two natural history cohorts (LUMC and NCL) was comparable, while MMP-9 levels declined in the drisapersen cohort (p < 10^−4^ and p < 10^−9^ for LUMC and NCL cohorts, respectively, Fig. 3D).

**Figure 4:**
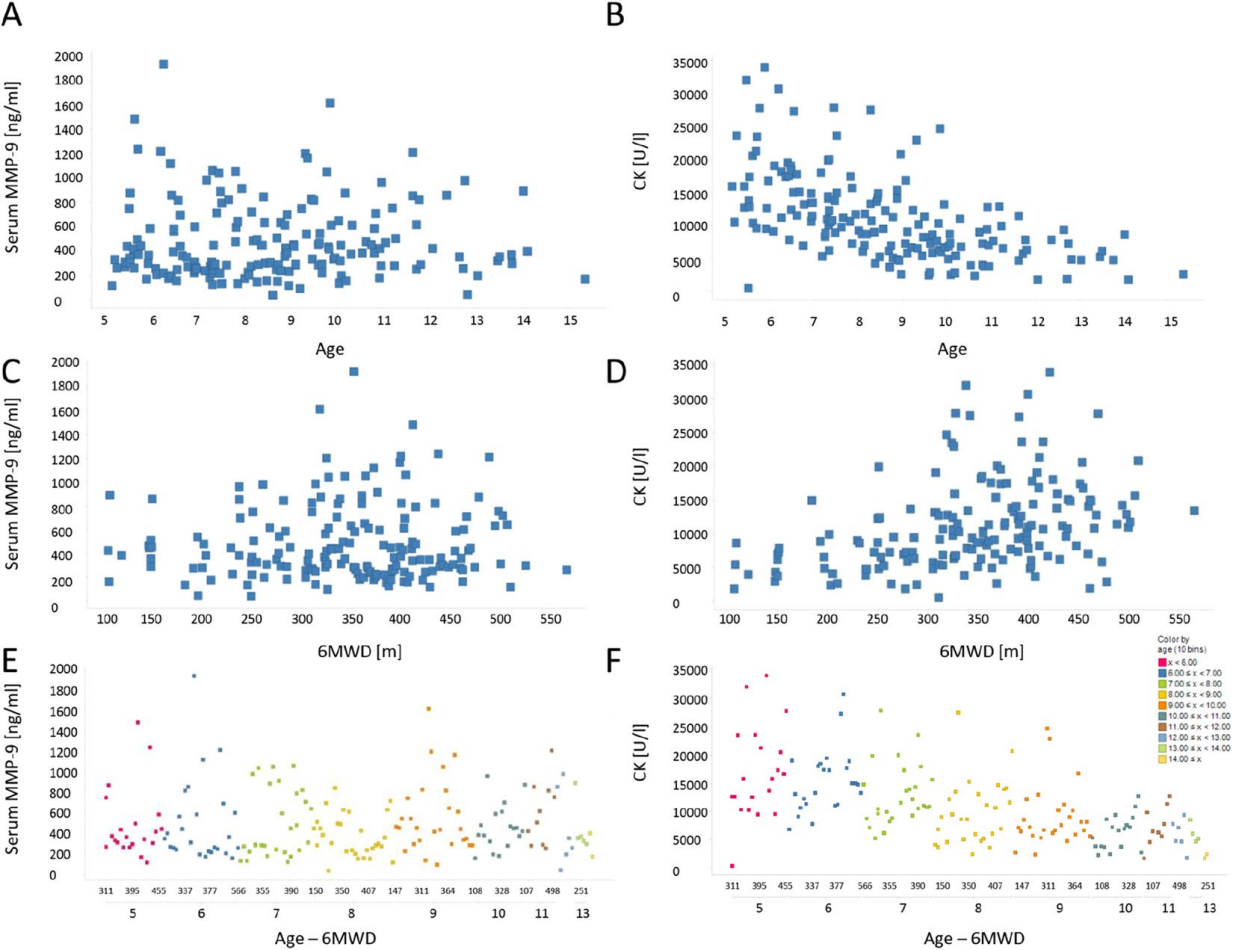
Relationship between MMP-9, CK, age and 6MWD at baseline in the phase 3 study (NCT01254019 - DMD114044). (**A**,**C**,**E**) Scatter plots showing the relationship between MMP-9 serum levels with age, 6MWD and the combination of age and 6MWD data. (**B**,**D**,**F**) Scatter plots showing the relationship between CK activity levels with age, 6MWD and the combination of age and 6MWD data. In panels E and F, color represents age windows as shown by the legend at the bottom-right and 6MWD are plotted for each age bin.

**Figure 5:**
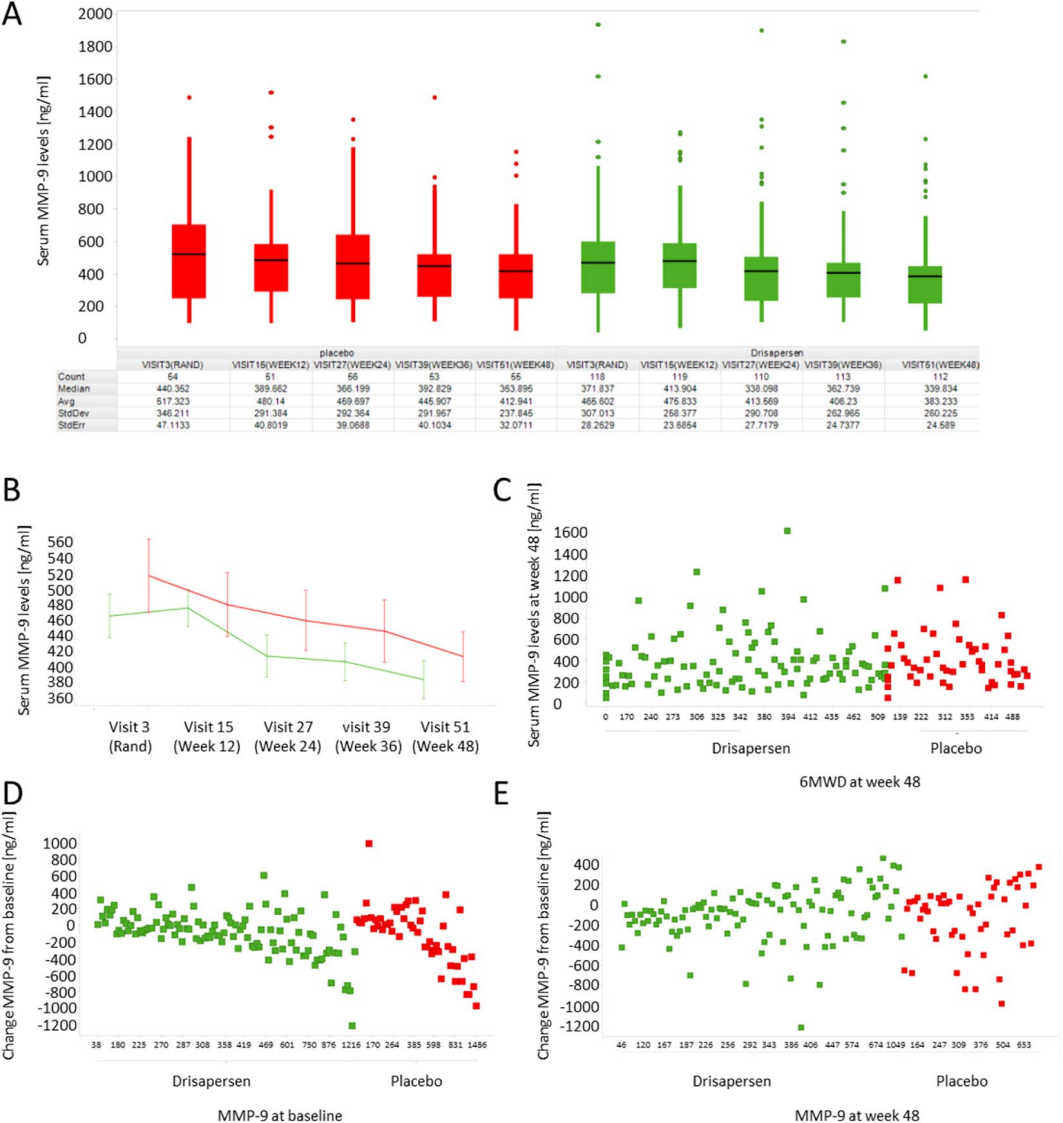
Effects of drisapersen on MMP-9 in the phase 3 trial (NCT01254019 - DMD114044). (**A**) Box-plot showing the MMP-9 levels at each visit for both placebo (red) and drisapersen (green) arms. For each visit the number of samples, MMP-9 median, average, standard deviation and standard error are given in table. (**B**) Line plot showing the progression of MMP-9 during the 48 weeks of the trial. The red line indicated patients on placebo, while the green line represents patients on drisapersen. (**C**) Scatter plot showing the lack of association between MMP-9 data and 6MWD at week 48. (**D**,**E**) Scatter plots showing the change in MMP-9 levels at week 48 (y-axis) compared to MMP-9 levels at baseline (**D**) and at week 48 (x-axis).

**Figure 6:**
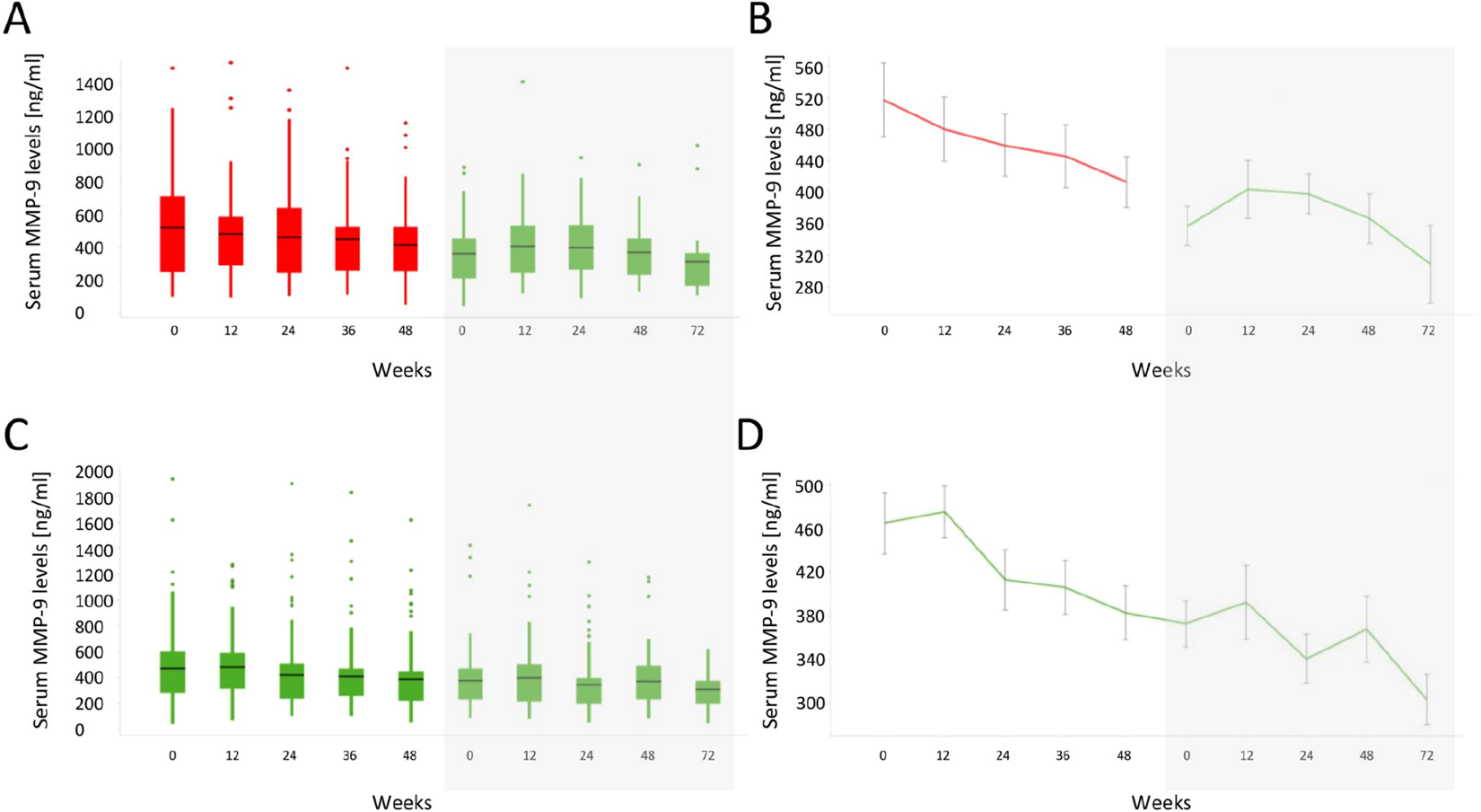
MMP-9 levels in the open label extension study DMD114349. (**A**,**C**) Box plots showing the concentration of MMP-9 in serum during the blinded phase (white background) and during the open label extension (grey background) for both placebo (**A**) and treatment (**C**) arms. (**B**,**D**) Line plots showing the mean MMP-9 serum concentration during the blinded phase and open label extension phase for both placebo (**B**) and treatment (**D**) arms. Data are presented as mean MMP-9 and error bars depict the standard error of the mean.

To validate the role of MMP-9 as predictive biomarker for dystrophin restoring drugs, we studied MMP-9 serum levels in sera obtained from the phase 3 study DMD114044 obtaining 841 data points. MMP-9 serum levels at baseline did not show significant associations with age or 6 minutes-walk test data (Fig. 4A,C,E). Creatine kinase (CK) activity levels were found to be reduced in older patients as expected (Fig. 4B,D,F). MMP-9 levels at baseline did not correlate with levels of muscle enzymes known to be elevated in DMD (CK and lactate dehydrogenase (LDH)), while CK and LDH levels were strongly associated (Fig. S3). There was a clear association between patient age and duration of the treatment with corticosteroids, however no evident relationship was present between MMP-9 levels and duration of corticosteroids treatment (Fig. S4). MMP-9 levels were found to be comparable between the placebo and treatment arms at baseline and for both groups MMP-9 levels significantly decreased during the duration of the trial (Fig. 5A,B). No association was found between MMP-9 titers and 6MWD at week 48 when the trial ended (Fig. 5C). The biggest reduction of MMP-9 levels over time were observed for those patients presenting with high MMP-9 levels at baseline, while higher MMP-9 levels at week 48 were originating from mild increase during the total trial duration (Fig. 5D,E). All patients participating to the placebo controlled trial were offered to participate to an open-label extension study where all patients were receiving drisapersen. MMP-9 levels were quantified in 576 samples obtained during the open label extension study and further decrease of MMP-9 serum levels was observed during the 72 follow-up weeks in both groups (Fig. 6A–D).

## Discussion

Duchenne muscular dystrophy is caused by lack of functional dystrophin, due to mutations in the *DMD* gene^23^. Several therapeutic approaches have been in development in the last few years ranging from stem cells, viral gene therapy and RNA targeting therapies such as exon skipping^18,26–28^. For all these approaches *in vitro* and *in vivo* proof of principle, mainly in the *mdx* mouse model, supported the clinical experimentation in DMD patients. For most clinical trials at least one muscle biopsy was requested to obtain proof of mechanism. Yet muscle biopsies represent an invasive procedure limited to a single muscle, thus it would be preferred to monitor disease progression and response to treatment in the least invasive possible manner. To achieve this, considerable effort has been placed to identify molecular markers which are differentially represented between DMD patients and controls, in blood derived fluids such as serum and plasma. This has the added benefit that, while a single piece of muscle from one muscle group may not be representative for the condition of all muscles in the body, the circulating nature of blood is considered to provide a snapshot of the state of the whole body (30–40% of which is skeletal muscle in healthy individuals). A few proteins and nucleic acids have been identified among which muscle specific creatine kinase (CK) is already known for long time^29^. CK is a very useful biomarker for the identification and diagnosis of various myopathies including DMD, however reduction of CK serum levels over time make this marker difficult to use as biomarker for DMD disease progression^30^; however it has been reported that CK decreases following therapeutic intervention by ataluren and gentamicin^31,32^. CK is the most known among the muscle specific markers which are released in circulation by muscle due to leaky sarcolemma, but many others have been recently identified or confirmed such as CA3, MDH2, TNNT3, TTN, MYOM3^33,34^. While these markers are of interest for diagnostic purposes and may help to better understand the muscle condition at a given time point, they remain of difficult interpretation as they all decrease as disease progresses; it is however possible that the extent to which they decrease over time contains information regarding disease progression. We have shown in the past that serum MMP-9 levels were higher in DMD patients compared to healthy controls and that serum levels increase over time in a small longitudinal cohort^24^. MMP-9 is already known in the Duchenne field due to its role in disease pathogenesis^35^ and for the ability to modulate the capacity of stem cell to correctly engraft in a dystrophic muscle^36^. MMP-9 has also been connected to heart disease^37,38^, tissue damage and cancer metastasis^39,40^ making it a non-specific marker, which is not appropriate to use as diagnostic marker for DMD. In this study we show that serum MMP-9 levels are elevated in DMD compared to healthy individuals by analyzing the serum of 172 patients and 32 healthy controls, providing a good but not excellent separation as assessed by ROC curves analysis. We elucidated that the inactive form is the most prevalent form in patients’ sera, probably due to inefficient MMP-9 activation in the extra-cellular space and quick uptake by blood vessels. Verification of the used ELISA kit (quantification range, precision, linearity, specificity and parallelism) and significant correlations with 2 independent techniques (antibody array and gelatin zymography) demonstrated that this method is in line with regulators guidelines for serum sample analysis. We report MMP-9 levels increase over time in 2 large independent longitudinal cohorts, and since DMD is a progressive disease, one could postulate that MMP-9 correlates with disease progression making MMP-9 a potential biomarker to monitor disease progression in Duchenne patients. This indication is also supported by a recent paper in which MMP-9 and Adiponectin were reported to be increasing over time in a relatively small cross-sectional cohort of DMD patients^34^. The association with disease progression remains however to be demonstrated by studying correlations between MMP-9 levels and clinical outcome measures. Interestingly, we observed in a group of 12 patients treated with drisapersen that high levels of serum MMP-9 significantly decreased with treatment over time. The observed reduction is significantly different compared to the 2 independent natural history cohorts, indicating that MMP-9 may have the potential to be used as predictive biomarker for treatment response in DMD patients. Nevertheless, it is important to consider that data points in the drisapersen study and natural history cohorts were not phased and that sampling frequency was lower in the natural history cohorts compared to the study involving drisapersen. To further study in depth the potential of MMP-9 as predictive biomarker we analyzed serum samples obtained during the phase 3 study (NCT01254019 - DMD114044) and the follow up open label extension study. More than 1400 data points have been analyzed showing comparable decrease of MMP-9 levels in both treatment and placebo arms during the blinded and open label extension phases, thus clarifying that the decrease in MMP-9 levels is not caused by treatment with drisapersen. This finding questions the validity of MMP-9 as predictive biomarker for response to therapy with dystrophin restoring drugs mainly because we did not observe the expected increase in the placebo group. It is possible that the shorter observational time compared to the natural history cohorts does not allow to detect an increase in MMP-9 over time or that MMP-9 levels at baseline in the natural history cohort were artificially reduced due to instability of MMP-9 during long term storage at -80 °C. This remains to be tested as literature reports show that plasma MMP-9 levels may or may not be affected by long term storage^41,42^.

Analysis of other serum biomarkers as exploratory outcome measures in large placebo controlled clinical trials will be important to verify/falsify our findings and ascertain whether candidate biomarkers can qualify as biomarker for disease progression and treatment response in DMD patients.

## Methods

### Study participants

DMD Natural History cohorts: Patients involved in the study were followed up at the Royal Victoria Infirmary, Newcastle upon Tyne (United Kingdom) (referred throughout the article as NCL), at the Leiden University Medical Center, Leiden (The Netherlands) (referred throughout the article as LUMC) and at the Dubowitz Neuromuscular Centre, UCL Institute of Child Health, London (United Kingdom) (referred throughout the article as UCL). Sixty-six DMD boys (38 boys from NCL and 28 boys from LUMC) participated in the longitudinal study (median age 10.8 years, age range 4.6–25.3 for NCL, median age 10.8 years, age range 4.7–19.4 for LUMC) involving blood sampling at different time points (Supplementary Table 1). Two to five serum samples per individual patient were collected. All patients included in the study were classified as DMD according to clinical examination, absence of dystrophin in the muscle biopsy assessed by either western blot or immunohistochemistry and frame disrupting mutation in the *DMD* gene confirmed by genetic testing. NCL also provided healthy control samples from boys without significant medical disorders, who were not on any systemic medication. The study was approved by the medical ethical commission of the Leiden University Medical Center and by the research ethics committee of the Institute of genetic medicine of the university of newcastle prior to commencement according to the principles set out in the WMA Declaration of Helsinki. Informed consent forms were obtained from each subject.

Open label study with drisapersen: twelve DMD patients with 51 flanking deletions for which the reading frame would be restored by exon 51 skipping, participating the in the open-label, phase I–II study dose-escalation study (NCT01910649) received weekly subcutaneous injections of 0.5–2.0–4.0–6.0 mg/kg of body weight (with 3 patients receiving each dose) drisapersen for 5 weeks. Subsequently, the patients were off treatment for a period of 9 to 47 weeks before entering the open-label Phase II extension trial (PRO051–02, study 114673), in which all patients received drisapersen by weekly subcutaneous injections of 6.0 mg/kg for 72 weeks, followed by 6 mg/kg/wk intermittent treatment (8 weeks treatment, 4 weeks no treatment). Individual subjects did not necessarily receive all injections according to the schedule. The median age at that start of the extension study was 10.1 years (age range 5.9–14.3). Seven serum samples were collected at 0-12-24-48-72-96-120 weeks after the start of extension study baseline, with the exception of one patient for whom the sample corresponding to 120 weeks was not available. Patients treated with drisapersen were monitored at the University Hospital Leuven, Leuven (Belgium) and at the Queen Silvia Children’s Hospital, Gothenburg (Sweden). Serum samples were analyzed according to research consent provided.

Phase 3 randomized, double blind, placebo controlled study with drisapersen: 186 DMD patients with deletions amenable of exon 51 skipping were enrolled in the study. The study was single dose with patients receiving subcutaneous injections of 6 mg/kg of body weight for 48 weeks (NCT01254019, DMD114044). Serum samples were analyzed according to research consent provided. Sera of 183 patients were analyzed. The median age at the start of the study was 8.0 years (age range 5.0–15.0). Samples were obtained at 0-12-24-36-48 weeks after the start of the trial. Serum samples were analyzed according to research consent provided. Following completion of the DMD114004 study (approximately 1 month later), both groups that received drisapersen or placebo in the DMD114044 study, entered the DMD114349 study and all received drisapersen treatment. After week 48 in the DMD114349 the number of subjects with available serum samples and MMP-9 results is reduced and data at week 48 is available for approximately 60% of the subjects that entered the DMD114349. At week 24 of the DMD114349 study data is available for approximately 90% of the subjects. The total number of serum samples for which MMP-9 data have been analyzed in the phase 3 and open label extension studies was 1417.

### Protocol to obtain serum and plasma samples

Venous blood was collected in tubes containing either spray-dried or gel clot activator for serum collection. Tubes were inverted multiple times to ensure adequate mixing of blood and clot activator. Blood was allowed to clot for at least 30 minutes at room temperature and was stored on ice until further spinning. Coagulated blood was centrifuged at 2,850 *g* for 10 minutes. Serum was carefully removed, aliquoted and stored at −80 °C pending use. Plasma was collected from venous blood in tubes containing anti-coagulant.

### Determination of MMP-9 in human sera and plasma

MMP-9 levels were determined in serum samples diluted 100 times (unless they were above the upper limit of the quantification range and were then diluted 200 times) and in plasma samples diluted 40 times, using a commercially available enzyme-linked immunosorbent assay (ELISA) (human MMP-9 Quantikine ELISA kit, R&D Systems, cat. n. DMP900, Abingdon, United Kingdom). The assay is able to detect the 92 kDa Pro- (precursor) and the 82 kDa active MMP-9 forms. All measurements were carried out in duplicate according to the manufacturer’s instructions. The absorbance was read using a 96 well plate ELISA reader at 570 nm and 450 nm. The absorbance at 570 nm was subtracted from the absorbance at 450 nm to correct for optical density imperfections in the plate. Determination of MMP-9 levels in serum and plasma samples using antibody-based microarray system was performed as previously described^33^.

### ELISA kit verification

Verification of the human MMP-9 ELISA kit for the validation parameters described by the manufacturer was performed at Biomarin GCLP laboratory by 2 operators. Quantification range as determined by calibrators was between 0.312 and 20.000 ng/ml with a coefficient variation (%CV) between the duplicates below 10% and accuracy of 100 ± 4% to the corresponding nominal concentration. Low, medium and high concentration quality controls (QCs from R&D systems, lot number 1291897, 1291898, 1291899) with values of 2.15, 6.85 and 13.37, respectively showed inter and intra assay precision CV <10%. Dilution linearity of serum endogenously containing MMP-9 was demonstrated up to 1:800 dilution, with a recovery 100 ± 20% compared to the 1:100 dilution and a precision of the concentration for all dilutions of CV <20%. Serum samples were diluted 100-fold in Calibrator Diluent RD5-10, unless they were above the upper limit of the quantification range and were then analyzed following a 1:200 dilution. MMP-9 concentration measured for two non-DMD serum samples at 1:100 dilution in 6 experiments, had an inter-assay precision of CV <10%. Inter and intra assay precision in DMD samples had a CV <15% and <10%, respectively. Furthermore, we demonstrated parallelism between serial dilutions of MMP-9 standard and serial dilution of 2 non-DMD serum samples endogenously containing MMP-9 (1:100-1:16,000); the precision of the estimated concentration for all dilutions had a CV <30%. In addition the specificity of the ELISA kit to measure MMP-9 in the presence of drisapersen in non-DMD serum endogenously containing MMP-9 was demonstrated in spike-in experiments and no interference was observed. To test whether freeze/thaw cycles have an effect on MMP-9 serum levels, 2 control sera were frozen and thawed 10 times in a period less than 3 months and then MMP-9 was measured in duplicate. MMP-9 levels showed an accuracy of 100 ± 30% up to 8 freeze/thaw cycles.

### Gelatin zymography

A gelatin zymography kit (Cosmo Bio Co., LTD, cat. n. PMC-AK47-COS, Tokyo, Japan) was used to determine the gelatin degrading activity of MMP-2 (gelatinase A) and MMP-9 (gelatinase B) in sera of 29 DMD patients. Before performing the gelatin zymography, total serum protein concentration was determined with the BCA protein assay kit (Thermo Fisher Scientific, cat. n. 23225, Etten-Leur, the Netherlands). Serum samples were diluted 50-fold in distilled water and the concentrations were measured in triplicates according the manufacturer’s instructions. Final protein concentrations were adjusted to 2 µg/µl and 20 µg (10 µl) of total protein was mixed with 10 µl of sample preparation buffer, incubated for 15 minutes at room temperature and loaded onto the provided gel. Ten µl of the MMP marker and 5 µl of a protein ladder (Thermo Fisher Scientific, cat. n. 26616, Etten-Leur, the Netherlands) were loaded as controls. The electrophoresis was run in a XCell SureLock™ Mini-Cell Electrophoresis System (Life Technologies, Carlsbad, CA, USA) at 150 mA for 2 hours. Latent MMP forms were activated by incubation with a reaction buffer, the gel was stained with the staining solution provided by the kit manufacturer and scanned using an Odyssey infrared imaging system (Li-Cor Biosciences, Lincoln, USA). Band intensities were quantified using ImageJ.

### Statistical analysis

Since MMP-9 data were not normally distributed we used nonparametric tests. We used a Mann-Whitney U test to test for differences between baseline MMP-9 levels of patients treated with drisapersen (at the start of the extension study PRO0-51-02) and patients from Newcastle and Leiden pooled together. We used a univariate linear model with serum MMP-9 levels as dependent variable to test the effect of cohort (LUMC, NCL, UCL), corticosteroid treatment, age and clotting time (only available for some samples of the NCL cohort). Longitudinal data of Leiden and Newcastle were analyzed with linear mixed models to determine the effects of age at baseline and follow-up time on serum MMP-9 levels. The fixed effects were age at baseline and follow-up time, while patients were considered as random effect. Residuals were normally distributed according to the Kolmogorov-Smirnov and Shapiro-Wilk tests. To compare the DMD natural history cohorts with the cohort of patients treated with drisapersen, we expanded the linear mixed model including the factor cohort (LUMC, NCL, drisapersen) as well as the interaction between cohort and follow-up time. The fixed effects were age at baseline, follow-up time, cohort and the mentioned interaction, while patients were considered as random effect. For the analysis of only long follow-up data or only short follow-up data, we used the same model as when all data points were used. Analysis of MMP-9 data obtained during the phase 3 trial was performed using a linear mixed model with MMP-9 as dependent variable. Fixed effects were MMP-9 levels at baseline, treatment arm, visit, the interaction between treatment arm and visit and the interaction between visit and MMP-9 levels at baseline; given the balanced design we did not use random effects, instead we used a repeated covariance matrix with compound symmetry structure.

## Electronic supplementary material

Supplementary Information
