# Supplementary material for: Evaluation of serum MMP-9 as predictive biomarker for antisense therapy in Duchenne
Source: Sci Rep. 2017 Dec 20;7:17888. doi: 10.1038/s41598-017-17982-y (PMC5738430; doi:10.1038/s41598-017-17982-y)
Supplement: Supplementary file 1 — Supplementary Information [file 41598_2017_17982_MOESM1_ESM.pdf]

Evaluation of serum MMP-9 as predictive biomarker for antisense therapy in Duchenne

A. Lourbakos[1], N. Yau[1], P. de Bruijn[1], M. Hiller[2], K. Kozaczynska[1], R. Jean-Baptiste[1], M. Reza[3], R. Wolterbeek[4], Z. Koeks[5], B. Ayoglu[6], D. de Klerk[2], G. Campion[1], I. Zaharieva[7], V.D. Nadarajah[2], P. Nilsson[6], C. Al-Khalili Szigyarto[6], F. Muntoni[7], H. Lochmüller[3], J.J. Verschuuren[5], N. Goemans[8], M. Tulinius[9], E.H. Niks[5], S. de Kimpe[1], A. Aartsma-Rus[2,3], Peter A. C. 't Hoen[2], P. Spitali[2]*

Supplementary Info

Figure S1

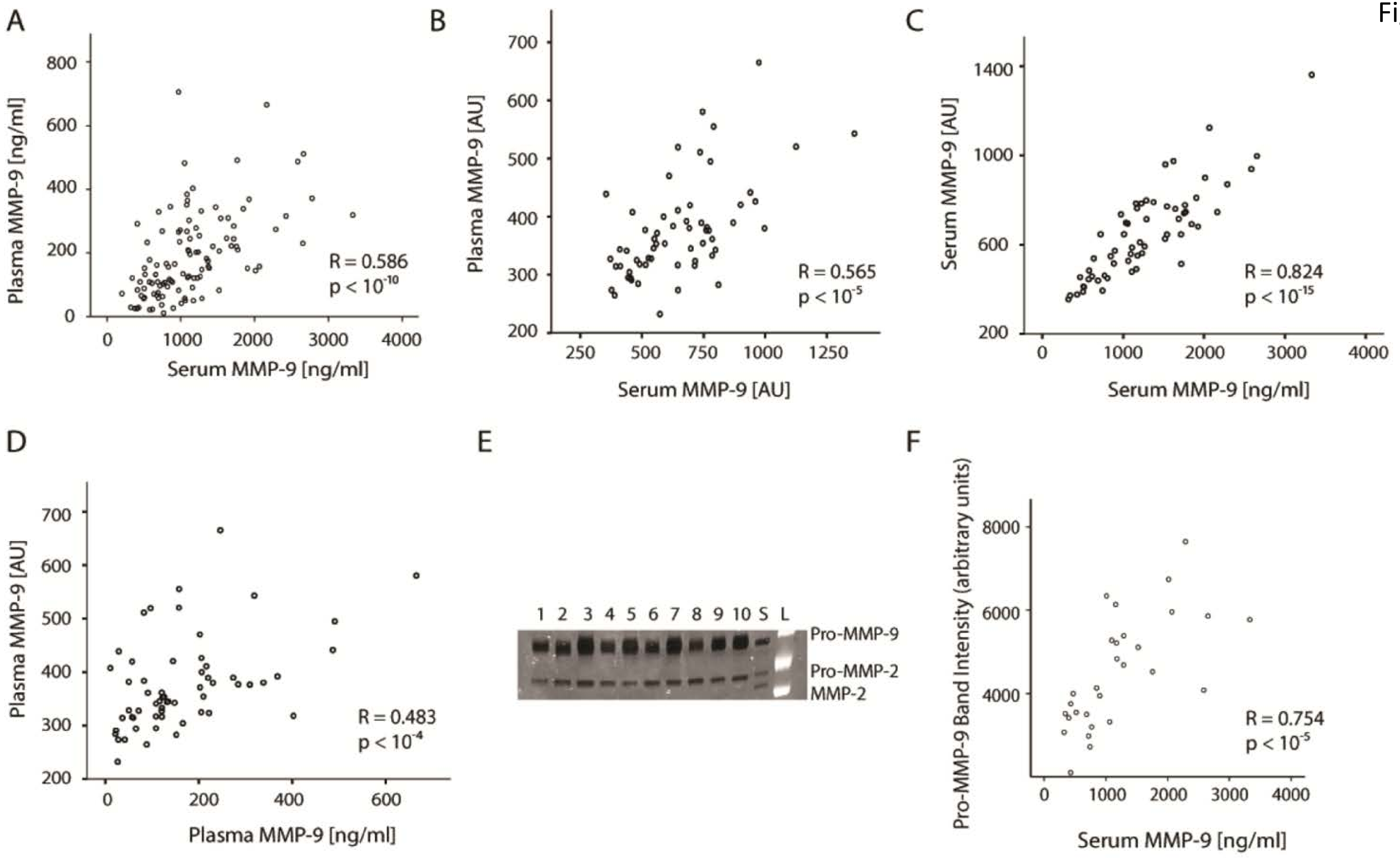
A
Plasma MMP-9 [ng/ml]
Serum MMP-9 [ng/ml]
R = 0.586
p < 10^-10
B
Plasma MMP-9 [AU]
Serum MMP-9 [AU]
R = 0.565
p < 10^-5
C
Serum MMP-9 [AU]
Serum MMP-9 [ng/ml]
R = 0.824
p < 10^-15
D
Plasma MMP-9 [AU]
Plasma MMP-9 [ng/ml]
R = 0.483
p < 10^-4
E
1 2 3 4 5 6 7 8 9 10 S L
Pro-MMP-9
Pro-MMP-2
MMP-2
F
Pro-MMP-9 Band Intensity (arbitrary units)
Serum MMP-9 [ng/ml]
R = 0.754
p < 10^-5

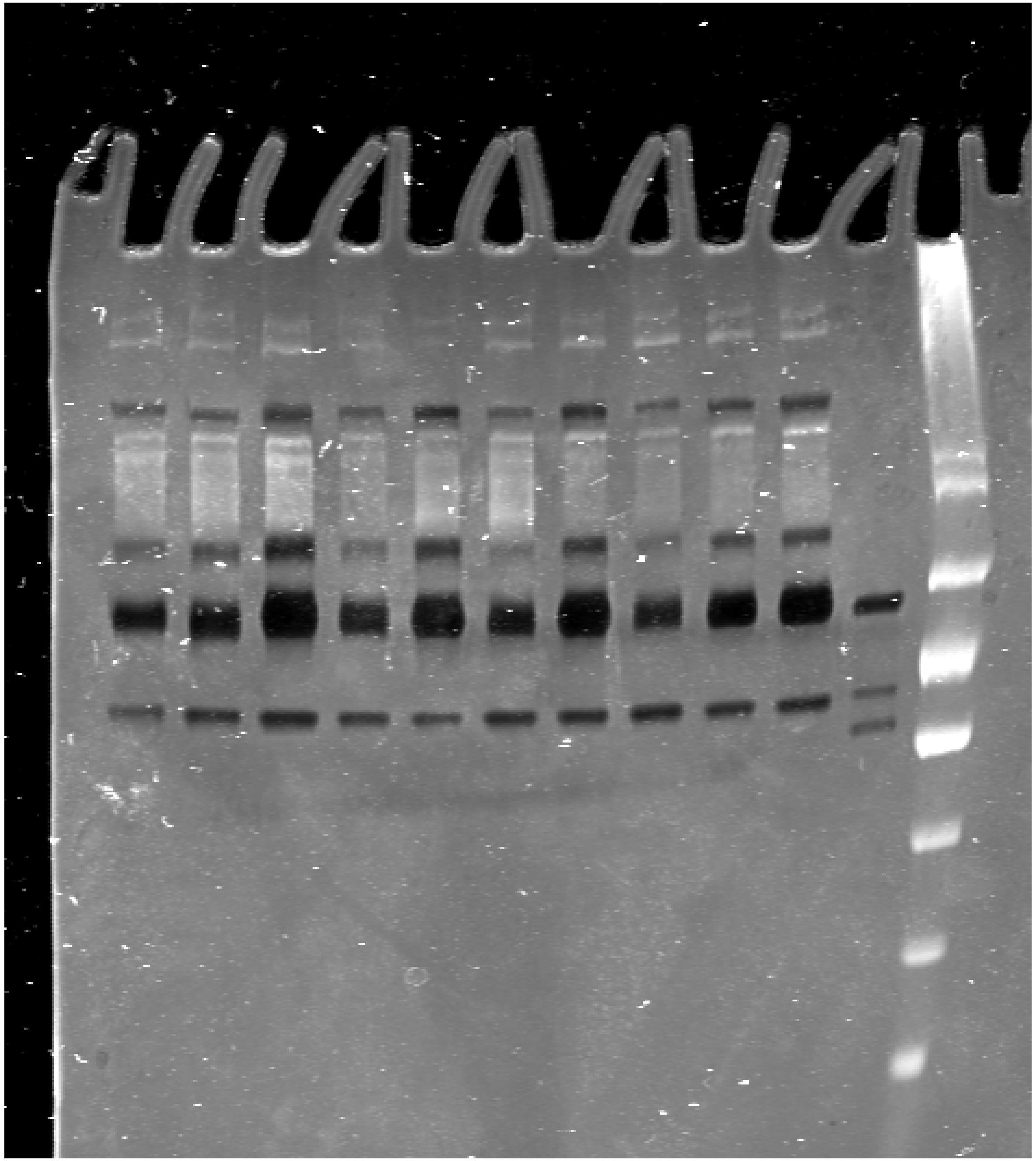

Full-length gel and of the image presented in figure S1 panel E

Figure S2

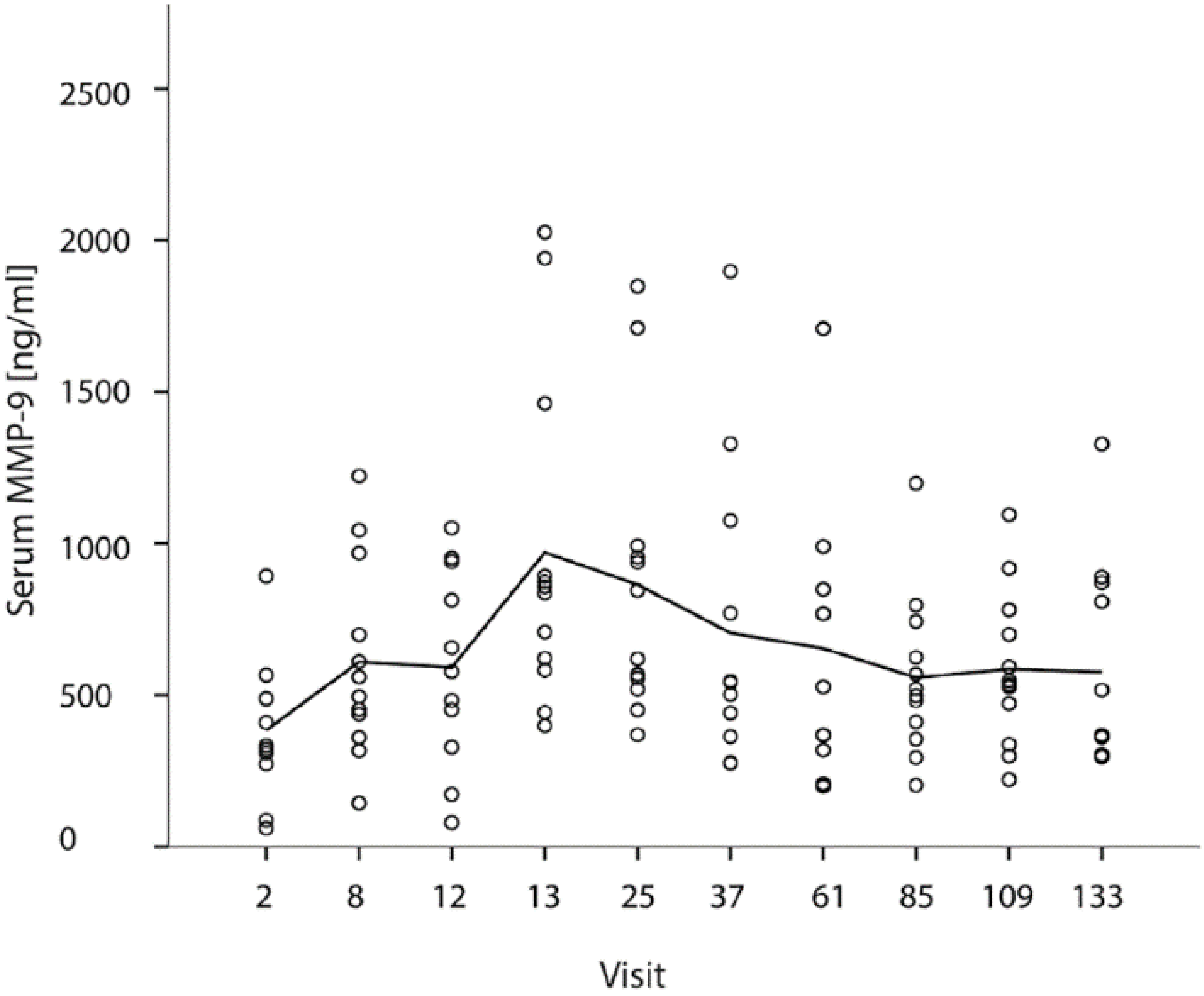

Serum MMP-9 [ng/ml]
2500
2000
1500
1000
500
0
2
8
12
13
25
37
61
85
109
133
Visit

Figure S3

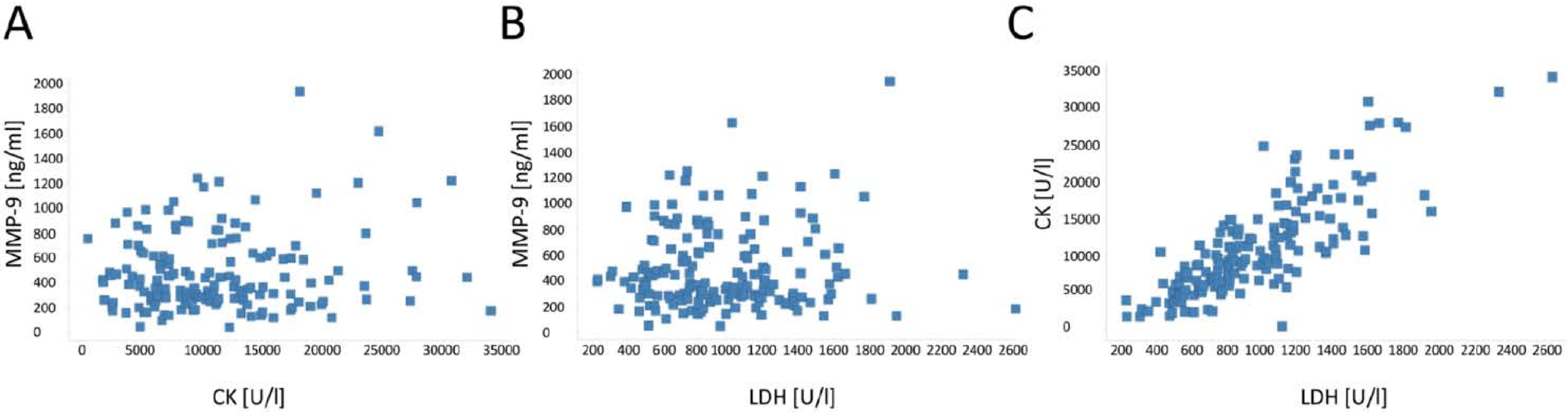
A
MMP-9 [ng/ml]
2000
1800
1600
1400
1200
1000
800
600
400
200
0
0
5000
10000
15000
20000
25000
30000
35000
CK [U/l]
B
MMP-9 [ng/ml]
2000
1800
1600
1400
1200
1000
800
600
400
200
0
200
400
600
800
1000
1200
1400
1600
1800
2000
2200
2400
2600
LDH [U/l]
C
CK [U/l]
35000
30000
25000
20000
15000
10000
5000
0
200
400
600
800
1000
1200
1400
1600
1800
2000
2200
2400
2600
LDH [U/l]

Figure S4

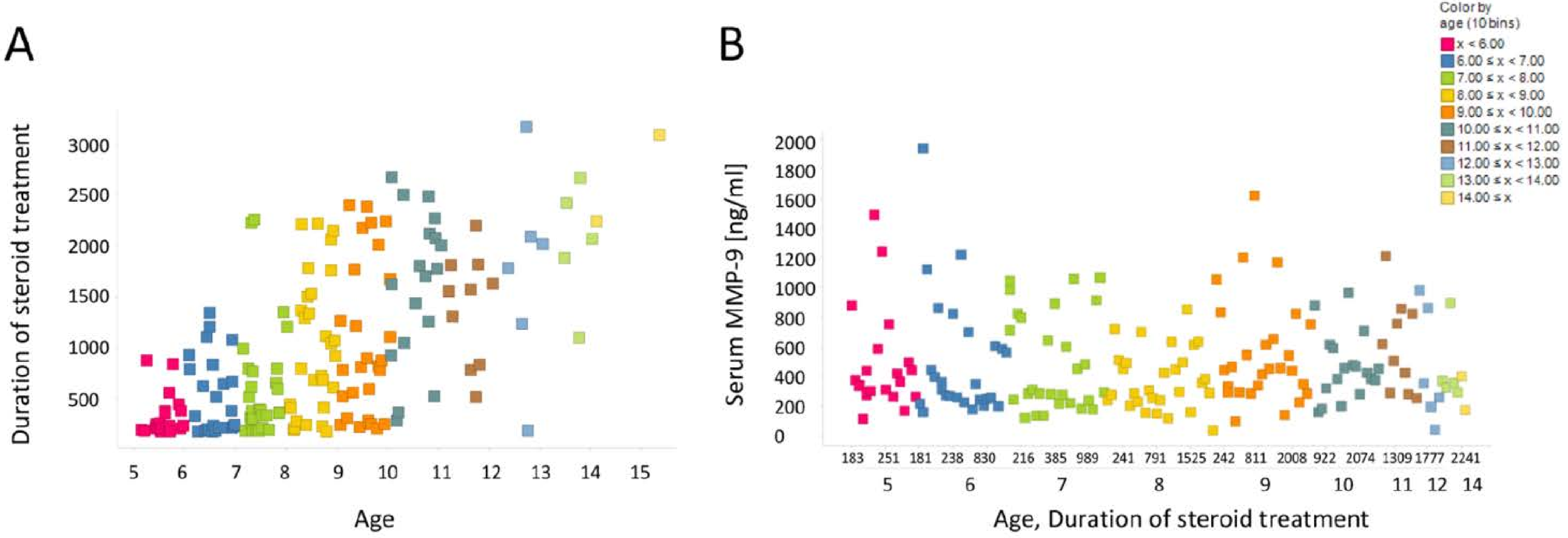
A
Duration of steroid treatment
3000
2500
2000
1500
1000
500
5 6 7 8 9 10 11 12 13 14 15
Age
B
Serum MMP-9 [ng/ml]
2000
1800
1600
1400
1200
1000
800
600
400
200
0
183 251 181 238 830 216 385 989 241 791 1525 242 811 2008 922 2074 1309 1777 2241
5 6 7 8 9 10 11 12 14
Age, Duration of steroid treatment
Color by
age (10 bins)
x < 6.00
6.00 ≤ x < 7.00
7.00 ≤ x < 8.00
8.00 ≤ x < 9.00
9.00 ≤ x < 10.00
10.00 ≤ x < 11.00
11.00 ≤ x < 12.00
12.00 ≤ x < 13.00
13.00 ≤ x < 14.00
14.00 ≤ x

Table S1

Reference	Assay vendor	N healthy children	MMP-9 ng/ml (mean± SD)
Zocevic 2015 [43]	R&D	51	500 ± 150
Głowińska-Olszewska, 2007 [44]	R&D	28	400.4 ± 204
Polanska, 2007 [45]	R&D	20	194.6 (121.13-231.54) median (25-75%)
Nadarajah 2011 [24]	R&D	12	73.2 ± 121 median ±IQR
Current report	R&D	32	256.7 ± 207.6

Table S2

Cohort	Patients	Time points
Newcastle	30	2
	2	3
	1	4
	5	5
Subtotal	38	95
Leiden	15	2
	9	3
	4	4
Subtotal	28	73
Drisapersen	1	6
	11	7
Subtotal	12	83
Total	78	251

Legends to Supplementary Figures and Tables:

Supplementary Figure 1. Technical validation of MMP-9 serum levels in DMD. **A-B**. Scatter plots showing significant correlations between serum and plasma MMP-9 levels in 111 serum and plasma samples obtained from DMD patients at the same moment using the ELISA assay (**A**) and in 60 serum and plasma samples as determined by antibody array (**B**). Serum levels are plotted on the x-axis, plasma levels on the y-axis. AU is arbitrary units. **C-D**. Scatter plots showing significant correlations between MMP-9 levels measured by ELISA and antibody array in 60 serum (**C**) and plasma samples (**D**). Each dot represents an independent patient sample. ELISA measurements are plotted on the x-axis, antibody array measurements on the y-axis. Spearman correlation was used to test whether significant associations exist. **E**. MMP-9 characterization by gelatin zymography. Example of gelatin zymography for 10 cases. Pro-MMP-9 and pro-MMP-2 are present in all samples, while the active forms are not detected. The control standard (S) shows both pro-active forms and the active form of MMP-2. L is molecular ladder. **F**. Scatter plot showing the correlation (Spearman) between serum MMP-9 values measured by ELISA (x-axis) and Pro-MMP-9 band intensity in the gelatin zymography assay (y-axis). No significant correlation was found between pro-MMP-2 band intensities and MMP-9 ELISA measurements. Each dot represents one of 29 DMD patients.

Supplementary Figure 2. Scatter plot showing serum levels of MMP-9 during the dose escalation study and the open-label extension study of study NCT01910649. MMP-9 levels are plotted on the y-axis while visits are plotted on the x-axis. Visits 2 to 12 are part of the dose escalating study, between visit 12 and 13 patients were not treated and this period varies between 9 to 47 weeks. Visit 13 represents the start of the open label extension study.

Supplementary Figure 3. Scatterplot showing the lack of association between serum MMP-9 and muscle leakage biomarkers. MMP-9 levels are plotted with CK (**A**) and LDH (**B**). A high correlation is present between CK and LDH levels for all patients involved in the phase 3 trial (NCT01254019, DMD114044) at baseline (**C**).

Supplementary Figure 4. Scatterplot showing the association between MMP-9 and duration of corticosteroid treatment. **A**. Plot showing the association between patients age and duration of corticosteroid treatment. **B**. Scatterplot showing the relationship between serum MMP-9 and duration of treatment with corticosteroids divided by age groups. For both panels the color represent age bins of 1 year starting from 5 years of age up to 14.

Supplementary Table 1. MMP-9 levels in healthy/ non DMD children reported in literature and in this manuscript.

Supplementary Table 2. DMD patients belonging to the natural history cohorts involved in the longitudinal study.
